# Targeted treatment of refractory primitive neuroectodermal tumor arising from an immature teratoma with crizotinib leading to a sustained response

**DOI:** 10.1002/ccr3.6779

**Published:** 2023-01-03

**Authors:** Benjamin M. Snyder, Alex H. Lion, Amy E. Helvie, Mark S. Marshall, Michael J. Ferguson

**Affiliations:** ^1^ Department of Pediatrics Indiana University School of Medicine Indianapolis Indiana USA; ^2^ Department of Pharmacy Riley Hospital for Children at IU Health Indianapolis Indiana USA

**Keywords:** crizotinib, Ewing sarcoma, PNET, precision oncology

## Abstract

Here we present a case of metastatic PNET which arose from an immature teratoma that was refractory to standard Ewing sarcoma chemotherapy. This PNET was determined to have elevated levels of ALK protein via IHC. The patient was treated with crizotinib on a palliative basis with a sustained response.

## Graphical abstract

When conventional therapy is exhausted in advanced malignancies, evaluation with next‐generation sequencing can identify targetable gene expression patterns like ALK overexpression in this PNET case, which led to a durable response with crizotinib.

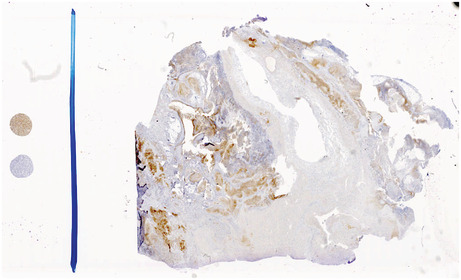

## 1 INTRODUCTION

Primitive neuroectodermal tumors (PNET) are rare tumors of aggressive phenotype with cellular appearance mimicking the differentiation of the central nervous system that can arise from germ cell tumors such as mature and immature teratomas. PNETs are grouped into two distinct morphologic entities, central and peripheral, based on histologic features consistent with CNS neoplasms in the central‐type and small round blue cells in peripheral‐type tumors mimicking Ewing sarcoma (EWS).^1^, ^2^, ^3^, ^4^ Given the overall rarity of PNETs, especially when arising from germ cell tumors, most data regarding their presentation, diagnosis, and treatment exist in several case series and reports. PNET has been demonstrated to primarily occur arising from the paravertebral space in the abdomen, ovaries, and uterus though these tumors do at times arise from both thoracic and abdominal soft tissue.^1^ Historically, treatment typically consists of a combination of surgery followed by traditional cytotoxic chemotherapy with or without radiation. Given the rarity of this disease, there is a paucity of genomic data regarding PNETs, leading to difficulty in conducting clinical trials with targeted agents. While peripheral‐type PNETs have been associated with t (11;22) translocation resulting in EWS‐FLI1 fusion gene typical of EWS‐type tumors,^5^ most PNETs arising from germ cell tumors resemble central‐type PNETs, which do not have identified genomic variants and lack chromosome 22 rearrangements.^4^ In the emerging era of targeted therapies, the ongoing identification of molecular targets remains crucial in the treatment of these rare tumors. Currently, molecularly targeted therapies remain salvage therapy in many types of malignancy, but the increasing ease of genomic, transcriptomic, and proteomic analysis is opening the door to the utilization of targeted therapies. Here, we present a case of metastatic PNET, which arose from an immature teratoma that was refractory to standard Ewing sarcoma chemotherapy. This PNET was determined to have high levels of ALK protein overexpression in the limited tumor sample, which led to utilizing targeted therapy with crizotinib on a palliative basis. This treatment resulted in a sustained partial response.

## 2 CASE PRESENTATION

**FIGURE 1:**
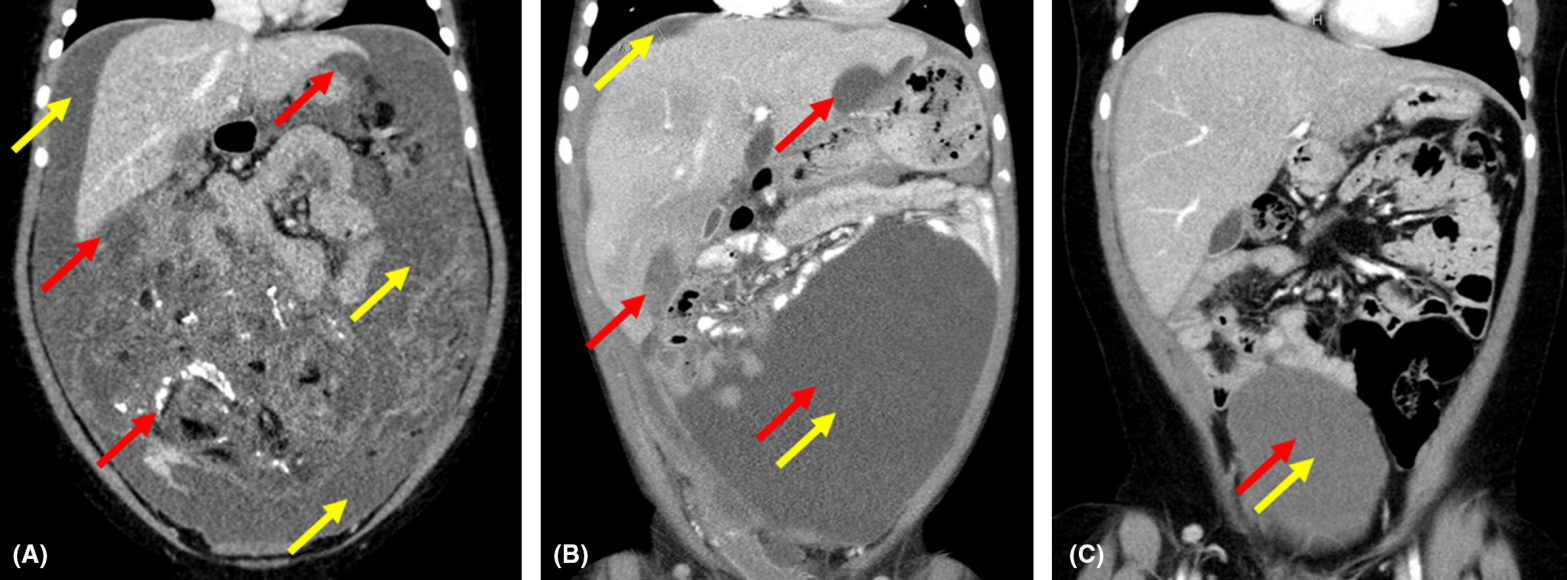
Side‐by‐side comparison of coronal CT imaging on diagnosis notable for significant ascites and large mass (A), relapse with recurrent ascites (B), and after 5 years on crizotinib therapy without malignant ascites and persistent necrotic mass (C). Red arrows represent areas of tumor, and yellow arrows represent areas of malignant ascites. When both red and yellow arrows are close together, this area represents known tumor that is obscured by malignant ascites in the pelvis.

**FIGURE 2:**
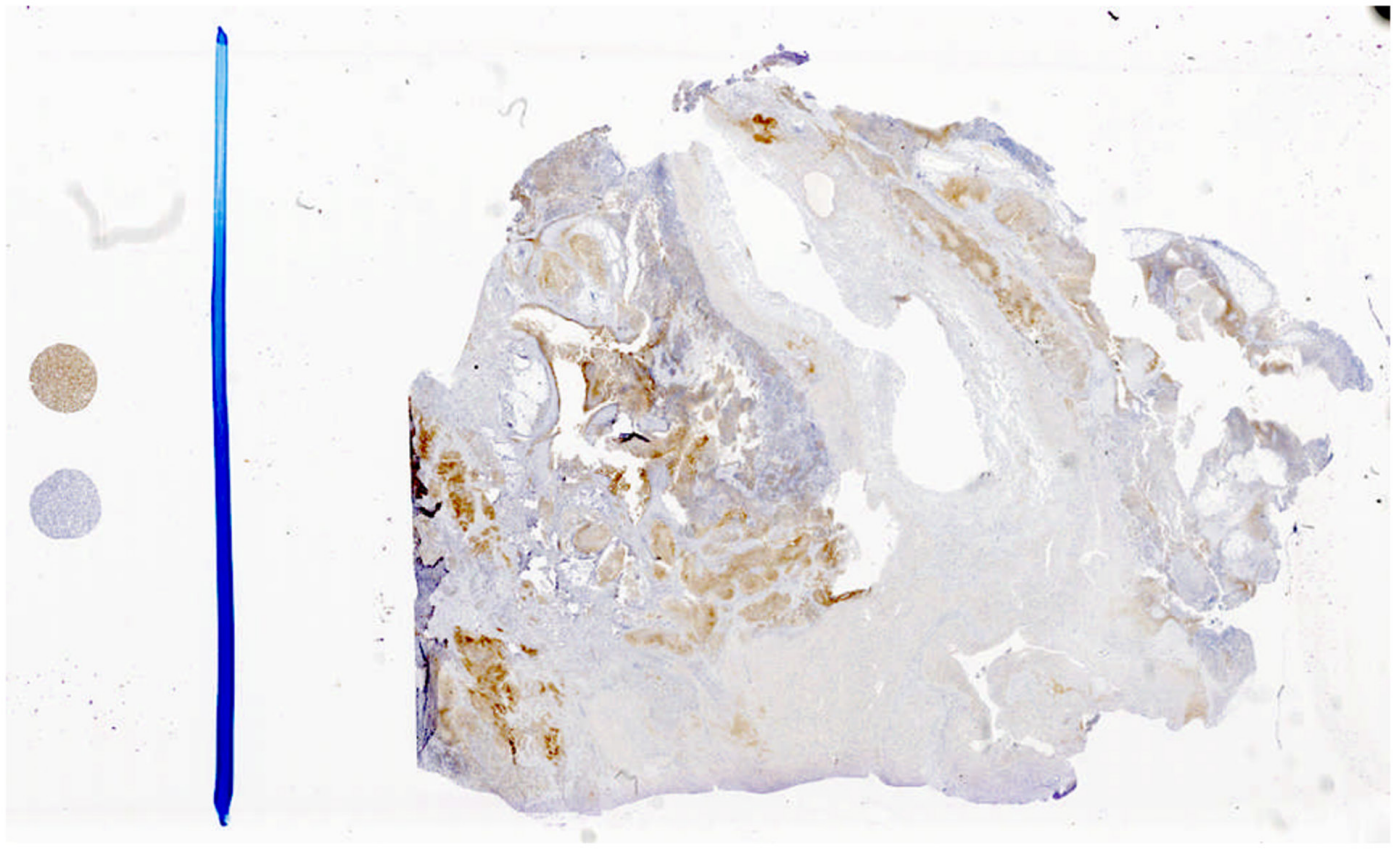
Anaplastic lymphoma kinase immunohistochemistry (IHC) was performed by Paradigm Cancer Diagnostics (PCDx). Appropriately reacting controls have been performed for all immunohistochemical (IHC) stains. The IHC stains were developed and their performance characteristics determined by Paradigm. Paradigm is certified under the Clinical Laboratory Improvement Amendments of 1988 (CLIA) as qualified to perform high‐complexity clinical laboratory testing. IHC scoring ranges from 0 to 4+ in intensity and 0% to 100% in percent noted in tumor sample. IHC for ALK demonstrated increased expression throughout tumor samples (2+ staining intensity in 30% of tumor sample). No pathologic mutations or gene rearrangements in ALK were identified on targeted sequencing by Ignyta.

Our patient initially presented in 2015 at the age of 5 years with approximately 2 years of worsening abdominal distension. At the time of presentation, a large right adnexal mass was identified by computed tomography measuring 15 cm × 11 cm × 13 cm with omental involvement and large‐volume ascites present (Figure 1). Initial imaging likewise revealed multiple soft tissue metastases to both the diaphragm and bilateral lung bases. Urgent resection was undertaken, and pathology from the primary tumor revealed high‐grade immature teratoma with areas of malignant primitive PNET. Tumor excised from the omentum revealed extensive replacement with immature teratoma with PNET features. Biopsies of peritoneal and diaphragmatic implants likewise revealed metastatic PNET with final histology consistent with central‐type PNET excluding EWS‐family features. Bone marrow biopsy at the time of diagnosis did not reveal that malignant infiltration and serum biomarkers including alpha‐fetal protein (AFP) and beta‐human chorionic gonadotropin (β‐hCG) were undetectable. Postoperative PET‐CT revealed remaining diaphragmatic, splenic, falciform ligament, omental, and peritoneal metastases. Given our institutional experience with high response rates in teratomas transforming to PNETs,^6^ chemotherapy was initiated with Ewing sarcoma‐like therapy with AEWS0031 Regimen B consisting of vincristine, doxorubicin, and cyclophosphamide (VDC) alternating with ifosfamide and etoposide (IE) 2 weeks after initial presentation and 10 days after initial surgical resection. Prior to week 9 of chemotherapy per AEWS0031, her course was complicated by a serious automobile accident leading to right femur and tibia fracture and a small subdural hematoma. These injuries led to a 4‐week delay in ongoing chemotherapy to allow for recovery. However, a PET scan obtained during this time of therapy revealed the significant overall reduction of disease in all areas. Once able to resume therapy, adjuvant abdominal radiation was planned to be given concurrently with week 13 treatment with VDC. However, she suffered multiple further complications with chemotherapy resulting from persistent febrile neutropenia, and *Streptococcus pneumoniae* bacteremia that led to radiation being delayed until week 15 of therapy. Our patient received a total of 34 fractions to the abdomen, lung, and adrenal gland for a total of 54.8 Gy. Follow‐up imaging postradiation and approximately 6 months after diagnosis unfortunately revealed significant disease progression in the abdomen and along the peritoneum with recurrent large‐volume ascites. Given the rapidity and extent of progression, a care conference was held where nontargeted therapy utilizing irinotecan and temozolomide was offered as palliative chemotherapy, but her parents chose not to pursue this due to its side effect profile. A consultation was made with our pediatric precision genomics team in order to pursue genomic characterization of this tumor to assess for any possible palliative therapeutic targets. Previously obtained tumor sample was sent to Paradigm PCDx for molecular characterization while discussions about hospice enrollment were ongoing. The Paradigm Genomic PCDx testing at that time was limited to NGS analyzing 500 genomic regions of interest including mutation, copy number variation, messenger RNA levels, gene fusions, and protein expression via immunohistochemistry (IHC). This test was negative for gene mutations or fusions within the panel. IHC testing was positive for anaplastic lymphoma kinase (ALK) protein (2+ staining intensity, 30% of tumor sample; see Figure 2) and neurotrophic tyrosine receptor kinase TRK/NTRK (80%), suggesting that either ALK or TRK/NTRK1/2/3 could be a targetable driver in the tumor. Gene sequencing failed to identify any mutations within the ALK coding region. The sample was tested for ALK, ROS1, and NTRK1/2/3 gene rearrangements by Ignyta and found to be negative. Early work has suggested that crizotinib, an oral inhibitor of ALK, has clinical activity in neuroblastoma patients whose tumors overexpress ALK protein in the absence of either ALK gene mutations or gene fusions.^7^ With few options remaining, crizotinib was selected as a therapeutic/palliative target based on the increased levels of ALK protein expressed in the patient's tumor (Figure 2). Therapy with oral crizotinib at 215 mg/m^2^/dose twice a day was initiated approximately 7 months after initial diagnosis and led to a reduction in the volume of multiple metastatic tumors. The patient remained stable for 4 years on crizotinib monotherapy without disease progression based on follow‐up imaging, and parents decided to discontinue therapy (Figure 1). The most recent follow‐up obtained in July 2022, 6 years after initiation of crizotinib and 18 months off therapy demonstrated ongoing disease stability without symptoms or evidence of progression (Figure 1).

## 3 DISCUSSION

In this case of PNET arising from an immature teratoma, multiomic analysis allowed for targeted treatment when traditional chemotherapeutic approaches lost efficacy. This is the first report of a PNET treated with targeted tyrosine kinase inhibitor (TKI) therapy. Treatment with crizotinib has shown efficacy in a variety of ALK mutant and rearranged tumors including non‐small‐cell lung cancer (NSCLC), inflammatory myofibroblastic tumors, and neuroblastoma,^8^, ^9^, ^10^ and now, there is emerging evidence of the effectiveness of ALK inhibitors in the treatment of tumors with ALK overexpression without mutation or fusion.^11^, ^12^ While PNET is a distinct entity from neuroblastoma, there are shared biological and molecular characteristics that make the finding of oncogenic ALK expression less surprising in the case presented here. It has been shown that embryonic neuroblasts and neural crest cells rely on ALK‐ and MYC‐pathway activation to maintain differentiation during embryonic development.^13^, ^14^ ALK mutation and even ALK‐wt overexpression have a significant impact on MYC‐pathway activation and tumorigenesis based on in vitro assessment.^15^ Specifically, in central‐type PNET, the pathologic appearance of neuroglial phenotype suggests similar pathway activation for neuroglial‐type differentiation as in neuroblastoma. Thus, a theory that ALK overexpression may be common in PNET tumors and simply has yet to be studied aligns with the known paucity of multiomic data for this tumor type. In addition to these biological features, ALK overexpression is an attractive target because of the tolerability of crizotinib and its ability to be combined with standard chemotherapy as in current chemotherapeutic trials for pediatric solid tumors.

As this case demonstrates, screening for targeted therapy options is vital when standard treatment options fail or are limited based on toxicity. This case demonstrates the clear benefit of targeted therapy in salvage scenarios as a sustained response was produced given the dependence of our patient's tumor on ALK‐pathway signaling. This case highlights the possibility that oncogenic protein upregulation via transcriptome or proteome analysis is underassessed in many clinical scenarios and can lead to valuable treatment options. Interestingly, in our case, the prolonged use of crizotinib did not lead to TKI‐specific resistance despite the frequent emergence of resistance mechanisms with long‐term crizotinib therapy reported in both NSCLC and neuroblastoma.^16^, ^17^, ^18^, ^19^ As more ALK‐targeted TKIs such as lorlatinib, which is being utilized in neuroblastoma clinical trials currently become available, moving from one ALK‐targeted TKI to another when resistance emerges could become easier similar to multiple‐tiered TKI use in chronic myelogenous leukemia (CML).^20^, ^21^ Given the rarity of this case, having further treatment options in the event of recurrence is helpful as crizotinib therapy may lose effectiveness. In summary, this case represents the first known use of crizotinib to target oncogenic ALK overexpression in central‐type PNET arising from an immature teratoma and highlights the importance of obtaining early molecular analyses in these rare tumor entities to determine novel treatment options.

## AUTHOR CONTRIBUTIONS

BS, AL, MM, and MF all have made substantial contributions to the conception and design, acquisition of data, and analysis of the data. BS, AL, AH, MM, and MF all have been involved in drafting the manuscript or revising it critically for important intellectual content, given final approval of the version to be published, and agreed to be accountable for all aspects of the work in ensuring that questions related to the accuracy or integrity of any part of the work are appropriately investigated and resolved.

## FUNDING INFORMATION

Authors with no other current funding for this research.

## CONFLICT OF INTEREST

We have no conflicts of interest to declare.

## ETHICAL APPROVAL

MM and MF were co‐investigators on P50HD090215 NICHD grant that expired in June 2022. The authors declare that there is no conflict of interest that could be perceived as prejudicing the impartiality of the research reported. Ethics approval was not required as this is a case report.

## CONSENT

Written informed consent was obtained from the patient's father to publish this report in accordance with the journal's patient consent policy.

## Data Availability

Data pertaining to the case report are available upon request.
